# Citizens' views on sharing their health data: the role of competence, reliability and pursuing the common good

**DOI:** 10.1186/s12910-021-00633-3

**Published:** 2021-05-18

**Authors:** Minerva C. Rivas Velarde, Petros Tsantoulis, Claudine Burton-Jeangros, Monica Aceti, Pierre Chappuis, Samia Hurst-Majno

**Affiliations:** 1 Faculty of Medicine, Institute Ethics History Humanities, University of Geneva, CMU/1 rue Michel Servet, 1211 Genève 4, Switzerland; 2 Department of Oncology, Geneva University Hospital, Rue Gabrielle Perret-Gentil 4, 1205 Genève, Switzerland; 3 Département de Sociologie, University of Geneva, Bd. du Pont-d’Arve 40, 1211 Genève 4, Switzerland; 4 Institute of Sociological Research, University of Geneva, Bd. du Pont-d’Arve 40, 1211 Genève 4, Switzerland; 5 Oncogenetics and Cancer Prevention Unit, Geneva University Hospital, Rue Gabrielle-Perret-Gentil 4, 1205 Genève, Switzerland; 6 Faculty of Medicine, Department of Community Medicine, University of Geneva, CMU 1 rue Michel-Servet, 1211 Genève 4, Switzerland

## Abstract

**Background:**

In this article, we address questions regarding how people consider what they do or do not consent to and the reasons why. This article presents the findings of a citizen forum study conducted by the University of Geneva in partnership with the Geneva University Hospitals to explore the opinions and concerns of members of the public regarding predictive oncology, genetic sequencing, and cancer.

**Methods:**

This paper presents the results of a citizen forum that included 73 participants. A research tool titled "the mechanics of consent" was designed for this study. This tool is a table encouraging participants to reflect on social and research actors, types of data, and desired levels of control while sharing different types of data with different actors. Participants’ discussion that led to the completion of each table were audio-recorded, transcribed, and analyzed using thematic analysis.

**Results:**

The results are a compilation of responses from the mechanics of consent tool divided into two sections; the first presents quantitative results of collective responses regarding attitudes to consent to donate their data. The second section present qualitative findings emerged from the discussion amongst participants.

**Discussion:**

Choice and control of personal data is crucial for the public to be able to decide who and how to trust. Key information to be disclosed to potential research participants shall include information about potential risks and benefits; who will be accessing and using their data; as well as assurances that their choice will be respected. Furthermore, researchers ought to make sure they are trustworthy, by acting in a competent, reliable, and honest manner. Governance systems ought to be better equipped to address ethical issues raise by the growing presence of non-traditional research actors, consent of exchanges of data via digital devices and online activity such as social media and fairness of data trading. Finally, informed consent is one of the various elements that contribute to conducting ethical research. More needs to be done to strengthen governance and ensure adequate protection of research participants, particularly to address issues related to predictive health analytics.

## Background

Informed consent in research is critically important, yet its implementation raises questions and is widely misunderstood [1–4]. The transformative potential of predictive analytics in precision medicine is bringing fundamental changes to medical care, treatment and research. As areas of medicine such as predictive oncology advance, enduring challenges of informed consent for research and participation are more critical. Furthermore, data generation in our everyday life with digital devices, social media and online shopping, data trading alongside a growing presence of non-traditional research actors such citizen science initiatives, triggers complex informed consent tools, procedures and protocols. More needs to be done to document how consent forms are perceived across social groups, what information should be disclosed to potential research participants, how disclosures should occur, as well as reciprocity and accountability of research and when it is appropriate to waive individual informed consent via institutional review board (IRB) approval. This paper contributes to the understanding of how people consider what they do or do not consent to, and the reasons why within this contemporary data intensive context in Canton of Geneva, Switzerland.

Informed consent procedures, traditionally consisting of a one-off act of signing a document, are seeing some changes, towards interactive web-mediated platforms in which potential research participants can tailor their preferences [1–7]. Some of more prominent participant-controlled models such as meta-consent offer potential research participants a large variety of choice including deciding how and when they would like to provide consent, alongside the possibility of deciding how they would like to provide consent in the future [8–11]. However, the implementation of such models showed that consideration and minimization of the burdens imposed by vast amounts of information and details remain unaddressed [11], as well as, choice limitation due to system functionality [12].

Either way informed consent as a one-off act of signing hard copies or presented as procedures sensitive to individual preferences are likely to fall short in disclosing all relevant information to all types of data usage. In predictive oncology, for example, it would not be realistic to disclose all current and potential future use of genetic information and technology [13]. This shortfall is not likely to be remediated by offering either wider or more precise information to potential research participants because it would render the consent form unintelligible or impractical, and cannot anticipate data use cases that do not exist at the time of project development. Manson and O'Neill [3] on their analysis of informed consent in biomedical ethics examined this issue. They found disclosure particularly problematic as by itself it may not reach the intended audience; it could fail to communicate what is proposed, or which commitments are offered by those requesting consent. Furthermore, at times it may even fall short in truly reflecting whether consent is given or refused.

O'Neill [4] analyses the key role of trust and accountability in informed consent and advocates that focusing on trustworthiness may be more promising in legitimating consent. For O'Neill trust is understood as intelligent trust growing out of active enquiry. Intelligent trust relies on what those seeking consent say, the truthfulness of their claims, their reliability of undertaking what they said they were to do, and keeping their commitments. Secondly, accountability serves trust by providing useful evidence for placing trust intelligently. O’Neill [14] further clarifies that trust is not a blank check: we give our trust when we believe that an agent will do a specific thing in a manner that is competent, reliable, and honest. This explanation is very relevant when reflecting about general consent. Perhaps, understanding responsible acceptance of trust for research actors, alongside adequate systems of accountability better serve ethical research practices than posing unrealistic demands on informed consent procedures that might ultimately undermine its significance. Brown Trinidad [15] claimed that trust between research and research subject is central to data sharing and the development of consent models. Furthermore, Anker et al. [16] found that the range of choice and control research participants might have makes little difference in their likelihood to consent for data sharing if there is a lack of trust on either research actors or tool use to record their consent preferences. Trust however does not eliminate the need for consent, but legitimizes it. Eyal [17] pointed out that trust is necessary for people participate in medical research.

In this article, we raise several questions regarding how people consider what they do or do not consent to and the reasons why. A citizen forum study was conducted for the University of Geneva in partnership with the Geneva University Hospitals to explore the opinions and concerns of members of the public society regarding predictive oncology, genetic sequencing, and cancer. This article presents the findings of one data stream of this mixed methods study that looks at informed consent in research.

## Methods

### Study design

The citizen forum was conducted by the University of Geneva in partnership with the Geneva University Hospitals to understand citizens’ expectations and fears regarding precision oncology. Citizen forums are a well-suited method to collect a wide range of perspectives in contested ethical areas [18, 19]. This format also named deliberative forums or democratic forums aimed to encourage effective participation and collective reflections on possible solutions to the ethical and social dilemmas associated precision oncology [20]. Fung [21] uses the terminology "minipublic" in an educative form, including all of the diverse voices, taking each other’s’ claims, explanations, reasons, proposals, and argument seriously. Street and al. [22] underline the “ideal conditions” to sustain citizen deliberation during citizen forums, i.e. diversified recruitment, independent oversight, moderation. Our study considered a representative sample, a comfortable ambiance, the adaptation to different levels of literacy, the expression of private and contrasted opinions through anonymization, a sensitivity to social and ethical consequences of personalized medicine/oncology. A comprehensive description of this citizen forum design and implementation has been documented on Aceti et al. [23].

Each of the four forums included two session of 2 h and 30 min placed 7 or 10 days apart. An average of 18 citizens participated at each forum and its 2 sessions. The programme was the following:

- One of the authors delivered a short presentation about precision oncology. Then, participants were formed into groups of 5 or 6. Authors of the study acted as group moderator or observers. In small groups, participants discussed the expected benefits and risks in genetic analysis. Finally, all forum participants collectively discussed main outcomes.
- The second activity was a debate on risk perceptions and their usefulness for medical decisions. First by small groups moderated by the authors, then main outcomes were discussed collectively.
- The third activity was an individual written-exercise and then group work on the rights and duties related to the transmission of personal genetic information. First by small groups moderated by the authors, then main outcomes were discuss collectively.

The second session included three new blocks of activity.

- The first activity was a collective review of previous session.
- For the second activity, one of the authors delivered a short presentation about the benefits and challenges of research in molecular tumors and management of patient genomic data. Then participant discuss first by small groups moderated by an author, then main outcomes were discuss collectively.
- The third activity of the session was the implementation of ‘the mechanics of consent’ which will be further explained below.

This last exercise ‘mechanics of consent’ lead to stream of data presented on this paper.

### Designing the mechanics of consent tables

A research tool titled "the mechanics of consent" (Table 1) was designed to allow participants to reflect on different elements of informed consent in health research, predictive oncology, and personalized medicine. On this tool, one axis of the table listed a variety of social actors and the vertical axis listed potential uses of these data. Groups of 5 to 6 participants guided by facilitators explored scenarios created by the intersection of different actors and data uses, such as, would (a) I donate my data to the public hospital to have more precise information about health; (b) to improve my treatment; (c) to use my case to improve the treatment of others; (d) to discover a risk factor; (e) to analyse social determinants; (f) to sell to third parties, or exchanging data for a discount in care. The same logic was then applied to all research actors listed. The exercise was repeated, but this time including the option to withdraw consent at any time, for example “I would donate my data to the hospital for profit if I could withdraw them at any time.” Lastly, a third table recorded what type of data sharing should not be allowed under any conditions, e.g. donating data to put online freely.

**Table 1:** Mechanics of consent tool

I would donate access to	To receive advice	To improve care for me	To improve care for others	To do disease research	To do research outside of health	Making money
Public hospital						
Other public services						
Pharmaceutical companies						
Other public services						
Patient associations						
Other associations						
Me						
For everybody to put online						

The mechanics of consent exercise allowed participants to reflect on social and research actors, types of data, and desired levels of control while sharing different types of data with different actors. The mechanics of consent exercise lasted approximately one hour. In small groups of five or six people, a facilitator guided participants through the table of mechanism of consent. The facilitator would go line-by-line asking each participant whether they would grant consent to each actor for each of the potential data uses and asked participants to explain the reasons for his or her choice. After the participants discussed each scenario, facilitators manually recorded responses to indicate consensus agreement in favour or against data sharing, or lack of consensus.

### Participant selection

An open call was launched to participate in a citizen forum regarding predictive oncology and personalized medicine in the local newspaper (which has free distribution to all households) in the Geneva region in Switzerland including social media (Facebook, Twitter, and LinkedIn). This type of convenience sample allowed the research to be presented to a large proportion of the population in the region and provided equal opportunities to different types of people to enroll on the citizen forum. Those that responded to the advertisement were invited to complete an online questionnaire regarding attitudes towards ethical and social dilemmas associated with predictive oncology and personalized medicine, also including sociodemographic characteristics that enable researchers to model judgment small group for discussions [18]. Special attention was given to stratify these groups by age and sex so each groups would be heterogeneous, yet all groups were similar between one another. Limitations regarding socio-demographic heterogeneity are addressed at the limitation section.

### Human participant protection

Each participant received an electronic information sheet describing the purpose of the research and a written consent form was sign before the forum. Participants received a compensation of CHF 200 for their time, totaling 8 h over 2 sessions, and to offset any expenses related to commuting. This compensation was calculated based on the minimum hourly wage in the Geneva canton (CHF 25/h × 8 h). The rewards had the advantage of attracting participants from modest backgrounds and basic education, which sustain a socio economical diversified sample. This study received a review waiver by the Geneva County Research Ethics Commission (reference number 2019-00681).

### Analysis

The responses from all groups’ discussion were compiled using Microsoft Excel, the data were extracted from all tables and is presented in the following section. The groups’ discussion that led to the completion of each table were audio-recorded, transcribed, and analyzed using thematic analysis. A primary coder coded all data; independently and separately, a secondary-coder coded 10% of the data and fed it into the development and refinement of the coding. All data were collected in French and extracted quotes were translated into English. The researchers working in the data analysis understood both French and English.

Coding base conceptual analysis was based on close and repeated listening of the audio, reading the transcription, and analysis of trends reflected on the responses to the mechanics of consent tables. First, data was broadly coded, for example, with codes such as ‘control,’ ‘access,’ ‘freedom,’ ‘frameworks’ ‘protection,’ and ‘vulnerability.’ Secondly, these codes were grouped according to relationship and proximity. For example, "The hospital making profit from my data, it’s a yes, but it depends if making money might not add bias to the research conducted with the data”. This was seen as one of several arguments about the need for ‘protection’ from exploitation and areas in which ‘health governance’ shall ensure protection for health care users and data donors, and that ‘data trading’ was by itself not inherently negative and could enhance ‘public good’ if profits were reinvested in public services. Table 2 presents the list of codes and themes established.

**Table 2:** Codebook

Agency	Access and control
	Data sharing decision making factors
	Data bartering and trading
	Open sharing
	Freedom
Common good	Altruism
	Data profit for the common good
	Expertise
	Trust
Adequate regulation and governance	Differentiated data sensibility
	Frameworks
	Need for governance
	Nuanced institutions
	Uncertainties regarding data usage/sharing outside health
Risk and protections	Exploitation
	Restricting data profit
	Profit linked with mistrust
	Vulnerability and discrimination
	Anonymized does not equal full privacy protection
	Protection
	Mistrust
	Harm can occur even with anonymized and coded data

## Results

This section presents the participants selected for the study and their answers. The results are a compilation of responses from the mechanics of consent tables divided into two sections; the first quantitative results followed by qualitative findings emerged from the discussion amongst participants.

### Participants recruited

A total of 110 individuals responded to the advertisement. Out of the 110, 73 participants completed the questionnaire and registered; they were aged between 18 and 78 years old, with 46 women and 27 men from various socio-economic profiles. 63% of participants attained a higher education level; the sample included students, people active in the workforce, and retired. Table 3 shows participants’ characteristics.

**Table 3:** Participants’ characteristics

	Citizen (N = 73)	
*Age*		
[18–30]	17	23%
[30–40]	11	14%
[40–50]	12	16%
[50–60]	14	18%
[60–70]	11	16%
[70–80]	8	12%
*Gender*		
Female	47	64%
Male	26	36%
Unknown	3	
*Education*		
Compulsory education	3	4%
Professional school, trainingship	10	14%
High school	8	11%
University	46	63%
Other	3	4%
Unknown	3	4%

### Collective response rates: attitudes to consent to donate their data

**Fig. 1:**
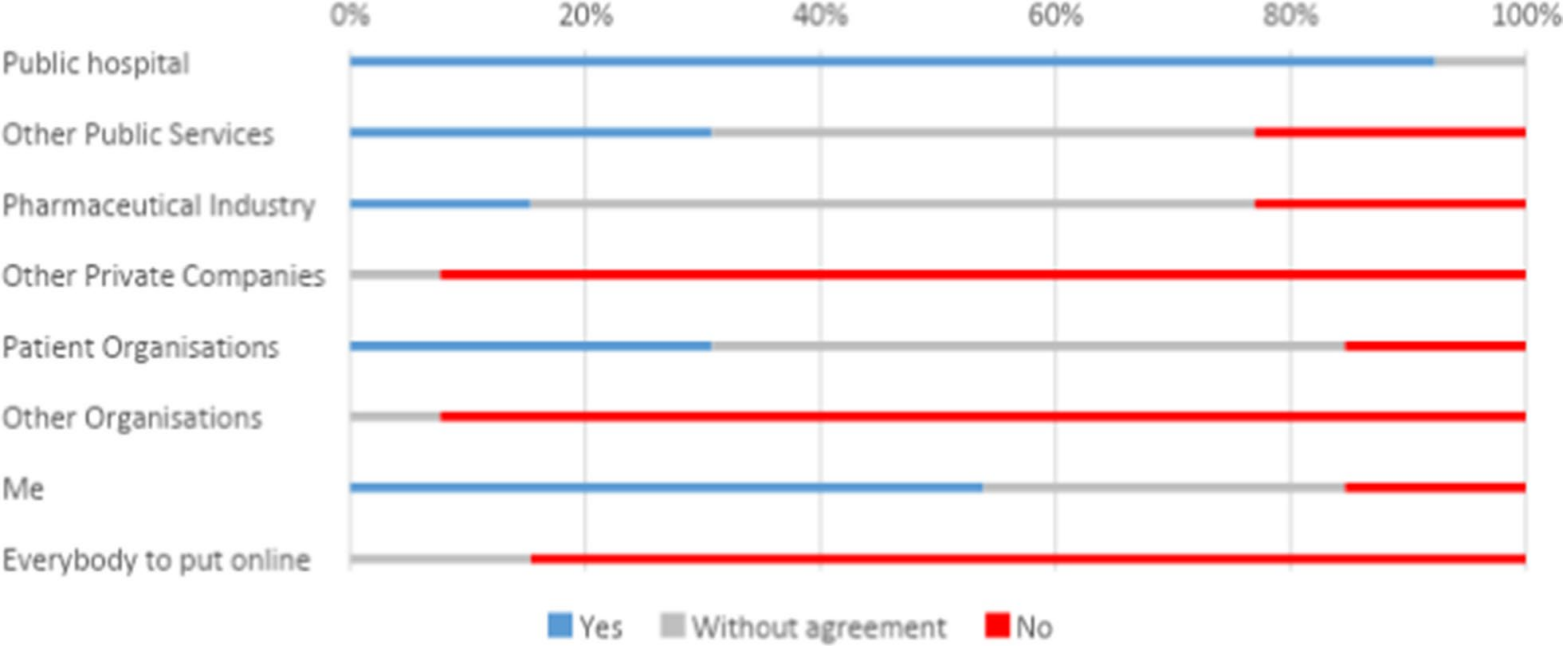
Answers to the statement ‘I would donate my data to do disease research’

We listed a series of potential actors in the mechanism of consent tool, namely public hospitals, other public services, pharmaceutical companies, patient associations, other associations, the public and the individual participant. Overall participants tend to be positively inclined towards donating their data to a public hospital. Figure 1 shows the results of the proposal ‘I will donate my data to do disease research’: an average of 92% of collective responses across participants groups would donate their data to a public hospital, all types of listed usages presented including improving information, their care, and research within and outside the health sector receive the same percentage of acceptance. The only category that participants were less likely to accept was donating their data. This applied to all listed actors, including the public hospital.

In addition, Fig. 1 shows that the next more positively perceived actors were oneself, most participants perceived positively this category with a small minority opposing it. This tendency remained for all data usage. Moreover, more than half of the participants would donate their data to patient associations, as long as the data were not used to generate profit. Overall, the least favorably perceived actors were private enterprises: none of the participants would donate them any data for any type of usage. Participants were also more negatively inclined towards open web-sharing with 90% of participants groups disagreeing with this type of sharing.

**Fig. 2:**
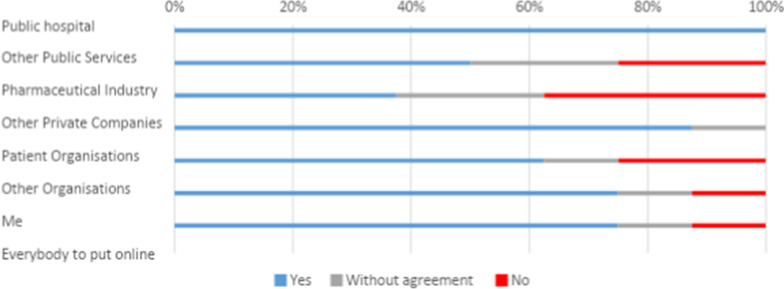
Answers to the statement ‘I would donate my data to do disease research, if I can withdraw at any point’

Donating data to the pharmaceutical industry for all type of usages tended to cause disagreement and if agreement was reached it was more likely to be against data donation. Participants’ views were more favorable if they could withdraw their data at any point. In Fig. 2, responses to the statement ‘I would donate my data to do disease research, if I can withdraw at any point’ show that having the possibility to withdraw made more than 10% of the participants change their mind and agree to share with pharmaceutical companies, in comparison to Fig. 1. Nevertheless donating to pharmaceutical companies still caused a large percentage of disagreement. The largest changes from Figs. 1 and 2 can be seen on self-profit: more people would agree to it if more control were gained.

**Fig. 3:**
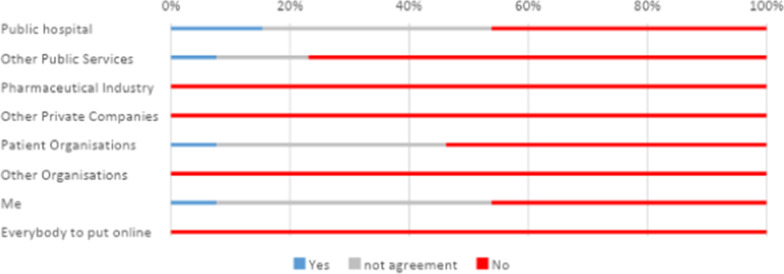
Answers to the statement ‘I will donate my data to making money’

Regarding data usage categories, participants tended to converge to a positive consensus about donating their data to receive medical or health-related advice, adjust their own treatment, improve health care for others and perform clinical research. However, this was only true if the data recipient was the hospital or themselves. The rest of the actors, namely other public services, patient associations, pharmaceutical companies, private companies, and open web sharing, were more likely to be denied sharing instinctively. The least accepted purpose for data use was to generate profit. Figure 3 ‘I will donate my data to making money’ shows that this type of usage was largely rejected across all data recipients listed in the exercise.

**Fig. 4:**
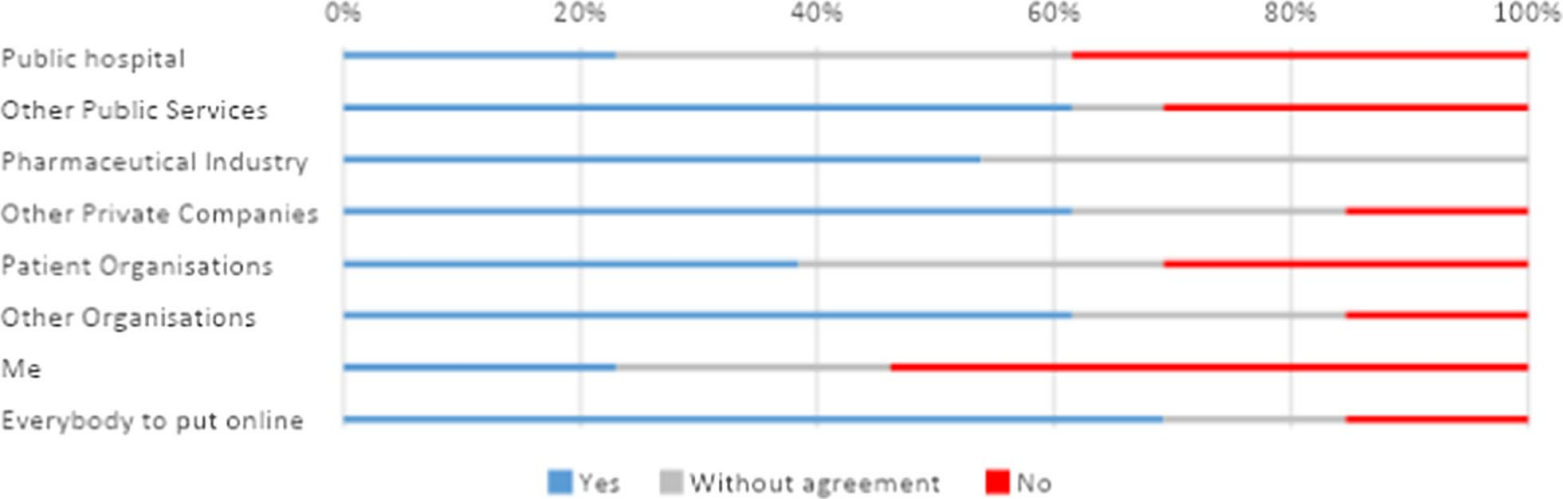
Answers to the statement ‘I will support banning donating data for profit’

Even for the hospital, if profit was envisioned the large majority of participants were either against data sharing or lack of consensus. Figure 3 shows profit making for patient associations and self-profit caused large disagreements among respondents. Furthermore, only 8% would agree to other public services and patient associations monetizing their data, the same number of participants would allow self-profit. It is also important that an only slight minority of participants support banning people from making money with their own data, thus in general, participants were favorable to banning profit making with the data to any other actors listed. The most divisive category was banning the hospital for making money, the idea of the hospital profiting with people’s data was problematic and most participants were inclined to ban such usage too. See Fig. 4.

The upcoming section will elaborate on themes that emerged from arguments presented by participants to explain their choices.

### Qualitative results: agency, protection and research for public good

This section presents the qualitative analysis on the discussion amongst participants while they were filling the tables. Four themes emerged: namely agency, risk and protections, adequate regulation and governance, and the common good. The analysis draws into the relationship among them and their overlap.

### Agency

The analysis exposes agency as a central argument to participants’ narratives: they would like to be able to have clear information and assess potential risk, foresee benefit and overall societal contribution of the research when deciding whether or not to donate their data. They also want assurance that their choice will be respected, and being able to withdraw their support according to their personal preferences and priorities. Their narratives illuminate various kinds of data that could be shared namely health records, biological samples, genomic sequence data, shopping habits, geographic location, and the like, while discussing the perceived risk that those might bear. Participants advocate that each person should be able to decide whom to share with, what type of data, for which purposes, and consequently what type of risk they would be willing to take. They believe that this opportunity is not always available to them. The following quote succinctly illustrates this point, hinting that is not the case right now and the sense of freedom that will accompany having such access and control.

‘It will be liberating to have access and control to our own data, it will not only be on the hands of somebody else’

FCIIIB_S_MECACONS

Participants’ narratives often use personal freedom to argue in favor of every individual ‘right’ to manage his/her health data, therefore, most participants were reluctant to deny people this possibility, including profiting from it.

It depends on what data you are sharing, why. That is freedom; we are talking about liberty… Otherwise were are not an authoritarian regime’

FCIB_P_5MECA1

They were also in favor of data bartering to gain a personal benefit like an insurance discount for example, thus being mindful of uneven power structures and risk of abusive exchanges. Participants overall expressed that at time of ill health despair might make people vulnerable to sharing and usage that participants deemed to be detrimental to the donor should be controlled and regulated.

### Misuse and protections

Participants see that all data they produce through their everyday life and use of mobile phones, shopping transactions, web search engines, social media and wearable technology provides information on who they are, revealing their behavior trends, health status and needs. The expressed concern that this information can be and has been misused in the past. They indicated that such information made people vulnerable among other to discrimination and exploitative market practices, and might trigger further mistreatment across vulnerable populations. They showed great mistrust towards profit driven data acquisitions, to participants such transaction amid exploitation. The upcoming quote expresses the concerns.

‘Those in poverty might sell their organs … they might face further exploitation if data becomes profitable, they will donate their data for money because they need it’

FCIB_S_5MECA1

A large majority of participants expressed their support for banning open-web sharing, citing high risk of re-identification, discrimination, misuse, and exploitation. A minority was skeptical about banning sharing in any circumstances. Those skeptical pointed out that consensual unrestricted personal data sharing already occurs outside a regulated context, for example sharing health data on social media via advocacy groups and the like. The same example was used by those who supported banning calling into question the lack of protection and potential for exploitation occurring on those outlets. They considered that allowing people to consent to give their data to profiting private companies or others not knowing if it had been misused has resulted in often exploitative issues and this cannot longer be the case, sharing restriction may be a way to combat this in their view. They stressed the lack of awareness and that little attention has been given to business model of such companies and risk for misusages of data in such circumstances.

“A: I will not share with private companies

B: …..such as Facebook, we already sharing a lot. And, I am not sure that is good for everybody”

FCIVB_S_2MECA

Participants believed anonymization is often presented as a protective shell though which no harm could occur, but believed that is no longer true. They argued that big data can precisely model and reveal health trends, needs, behaviors and other very sensitive information about sub- groups or even individuals and therefore anonymization is not enough protection in the era of big data. Harm can still occur and better protection is needed.

### Adequate regulation and governance

Legal and policy frameworks that ensure protection to individuals that might donate their data for research was key to the discussions. Perception on the efficiency of current frameworks and governance structure at times became a much-contested issue, while most participants expressed a high degree of confidence and trust in current legislation and its enforcement at the public hospital in Geneva. This degree of trust was not transferable to other institutions or jurisdictions particularly outside the country. Yet, some did not trust the current governance structure and recalled that sometimes legislation changes alongside political transitions in any given democratic country. Therefore, it was considered good to remain prudent and rather critical towards legislative frameworks and protection granted by them. The upcoming quote shows this skepticism.

A: I do not trust in democracy or legislation

We can’t, we see it in the (COUNTRY) now, things can change very quickly

So you do not trust in the politicians or democracy

B: No, I cannot trust in power elites

C: Me neither

FCIIIB_S_MECACONS

Participants expressed that some data are more sensitive than others are and an appropriate level of protection has to be granted accordingly. All participants perceived clinical data and particularly genomic data as highly sensitive, less agreement was found regarding other data that is relevant to health generated through everyday life, i.e. nutrition and shopping habits at the supermarket. Discussing different actors and data usage showed how participants perceived governance structures and the protections granted by them. As outlined above reluctance attached to profit and private enterprises including health related was often referred to as the lack of protection against exploitation. The section above shows how donating data to profit-seeking activities and entities was largely rejected, for, among other reasons, it was linked to uncertainty regarding legal protection, amid exploitation and immoral behavior. As discussion progressed, arguments unveiled that under efficient, just and transparent governance structures profit maybe positive. Profit was not seen as inherently negative and some recognized the potential to become a tool for public good as the following quote demonstrates.

If would donate (my data for profit making to the hospital) they make by reinvesting in more doctors and other personnel to take care of patients, if making money is immoral, I am not sure…maybe is more a solution as the hospital does not always have the resources

*FCIB_C_6MECA2*

Participants perceived that the protections granted to them in the online world regarding data transactions were either insufficient, limited, or non-existent. This included websites that might re-use the data for example genealogy sites or research actors publishing their databases. Although freedom online and efficient data use was highly valued, further protection and effective governance was required, as well as, better accountability on behalf of research actors asking for blank consent from data donors. This practice among other reasons, may contribute to uncertainty regarding donating data for research. Thus, doing and supporting research for the public good was a central point of discussion. The upcoming section outlines these findings.

### Public good

Moderator clarified that the decision to participate or not in research, should not have an influence in the quality of the clinical care. The possibility of personal immediate gain by getting advice about their own health concerns or potential improvement of their own health care was seen as favorable and tended to make people more inclined to consent to donate their data. However, in their narratives they pointed out that it was not a decision-making factor for most participants. Nor was the idea or prioritizing certain diseases or treatments, the ultimate decision-maker factor was whether or not the overall aim of the research was to maximize public good. Such ability to generate public good was systematically linked with public nonprofit institutions.

“A determinant factor is if we are talking about a public service, I’ll trust and share my data with a public hospital but not in a private one, that reassures me …we have frameworks, ethics protocols, conventions, legislation”

FCIIIB_C_2MECACONS

Participants’ narratives suggest a high degree of trust in public institutions, which are seen as generating public good. Trust was associated with accountability mechanisms and having checks and balances in place that were considered sufficient. However, trust did not always translate to perceived competence to best utilize data. Participants also judged professional competence and legitimacy to conduct high quality research separately. For example, patient associations tended to be perceived rather positively, however, they were not perceived as competent research actors, therefore participants would not donate their data to them to do research in any area.

‘Doing research is not the role of a patient associations’

FCIB_PT_5MECA1

Thus, competence to do research without trust was not sufficient either for person to accept to share their data. This was the case of the pharmaceutical industry, which tended to be negatively perceived. None of the participants would donate their data for research to the pharmaceutical industry, they acknowledged the crucial role that pharma has on health research and development, but such a recognition was not sufficient to be willing to donate their data for research. The pharmaceutical industry was largely distrusted, and perceived as exploitative, non-transparent, and pursuing wealth rather than the public good. The following quote illustrates this:

“A private company, is on its description, is an entity for profit, we have to see it like it, is there for a purpose to make money. We have mechanisms and frameworks to control that they do their job, they are not there to give us advice, but they are not there for the public good"

FCIIB_C_5MECACONS

Contributing to generate public good is in this case personal data donation to competent and socially accountable research actors that will provide a benefit to the public.

## Discussion

The results of this research highlight that it is critical to think of informed consent in a more comprehensive manner. As one of the various elements that lead to conducting ethical research, informed consent is a multi-party contract that builds upon reasonable disclosure of relevant information to data donors as well as responsible acceptance or rejection of trust on behalf of data recipients.

Participants in this study would like to receive sufficient information that allows them to assess potential risks and benefits when deciding whether to donate their data; they also want assurance that their choice will be respected. They would like to know about how their data will be used; who will be accessing and using their data; and the fundamental purpose of the research and its social impact. The results indicate that research actors perceived as professionally competent; generating public good; and whose work is being seen as conducted under effective governance translate into trustworthiness and likelihood to gain consent to use data for research. The discussion also noted that competency without generating public good or a lack of effective governance do not generate trustworthiness; thus, competency alone is not enough to enhance trust or gain consent as is shown in the discussions about donating personal data to pharma. Being perceived as trustworthy without being perceived as competent is also not enough to gain consent, as is demonstrated in the case of patient associations. These results echo Manson and O’Neill’s [3] account of trust, which explains that we give our trust when we believe that an agent will do a specific thing in a manner that is competent, reliable, and honest.

Researchers ought to make sure they are trustworthy, this means to accept or refuse trust when it is misplaced or built upon unrealistic expectations, meaning being clear to the public about their expertise and their limits. Refusing trust in such circumstances while providing an explanation that shows potential issues and a clear account of what they could be trusted with and what they cannot is a way to prove themselves as worthy. Gaining trust when they are not able to deliver in a competent, reliable, and honest way would be deceitful. A responsible acceptance to refuse trust shall facilitate constructive engagement between the general public and researchers.

Participants’ narratives show predisposition to view certain actors in a negative or positive light, this speaks about their lived experiences with this research actors that have made them to believe that some actors behave in a trustworthy manner. This often led to a deposit blank trust in some actors, like the public hospital for example, and mistrusting others in a similar manner. Trust or mistrust certain actors in an absolute manner means that we can make mistakes based in prejudgments rather than facts. These findings show that having choice and control over personal data is crucial for research participants. This is often portrayed in the literature as information self-determination, meaning the individual controls his/her data [24]. Today, self-determination is a right protected by domestic and international legislation across different jurisdictions [25]. Yet, participants expressed that they do not feel like they have control over their own data, even sensitive data such as their biomedical data. This raises two issues, first people presumably are not exercising their rights, or that opportunities to operationalize their choice are not present or known to them.

Furthermore, data generated through their everyday life via purchases, localization apps in mobile devices, social media, and the like were discussed at great length. The findings contribute to understand, compare and contrast citizens’ perspectives regarding consent for the use of data generated via digital devices, social media and other online activities such as online shopping, to genomic data. Participants questioned challenges regarding potential triangulation of data and past and future data misuse in social media, as well as a perceived lack of effective governance in the internet of things and inadequacy of protective measures such as anonymization. This last issue was discussed around the challenges of making data anonymous such as genetic data and concerns about harm (via group harm) that can still occur to individuals even if their identities are hidden. Discussions show that protection granted to the public should go beyond safeguarding anonymity, hiding only some personal identities, or solemnly names do not stop group harm. Data mining and modeling combined with raising computer power allows health trends, onset of illness, and risk behaviors to be predicted with great accuracy [26]. Legal frameworks in place today were not seen as robust enough to protect individuals if harm occurs.

The generation of profit is a very contentious issue. For some, profit may be welcome if it is reinvested to public services and ultimately generates public good, thus concerns remain about money generating bias or unlawful exploitation which made many reluctant to consent to donate their data for research if profit was envisioned.

## Limitations

The study was designed to identify concerns and reflect collectively on possible solutions to the ethical and social dilemmas associated with precision medicine, predictive oncology, genetic sequencing, cancer and biomedical research the Geneva region of Switzerland. Generalizability to national or international attitudes towards these issues as well as informed consent for research and medicine is unknown. Despite our best efforts to reach heterogeneous a sample women and persons with higher education were overrepresented on the study. An additional limitation is that presentation by experts increased familiarity with this topic and may not reflect the attitudes of uninformed citizens. Furthermore, this tool and its categories are not exhaustive. There are elements and interactions that we may have overlooked. There may even be research actors or data uses cases that do not yet exist but could be developed in the future. Nevertheless, the ‘‘mechanics of consent’ is a flexible tool that can be further developed and tested in response to evolving needs. Policy implication of the implementation of this tool are beyond the scope of this study.

## Conclusions

New data-mining techniques and knowledge of genetic factors and their impact on ill health offer very powerful predictions, providing opportunities for early interventions and highly personalized medicine. Thus, they also shed light to a broad set of ethical challenges. It also contributes to elaborate on the link of informed consent and trust [17]. The rapid progress in the field of predictive oncology, exacerbates the need to look at these unresolved issues of informed consent for research and medicine. This citizen panel showed that being perceived as professionally competent, generating public good, and being under effective governance translate into trustworthiness and likelihood to gain consent to use data for research. Choice and control of personal data is crucial for the public to be able to decide who and how to trust [27]. Key information to be disclosed to potential research participants shall include information about potential risks and benefits; who will be accessing and using their data; as well as assurances that their choice will be respected. Furthermore, researchers ought to make sure they are trustworthy, meaning they are responsible to accept or refuse trust when it is misplaced or built upon unrealistic expectations. Finally, informed consent is one of the various elements that contribute to conducting ethical research, more needs to be done to strengthen governance and ensure adequate protection to research participants particularly to address issues related to predictive health analytics.

## Data Availability

The datasets generated and/or analysed during the current study are not publicly available due to high risk of re-identification but are available from the corresponding author on reasonable request. Request for data access would be asses alongside the Ethics Commission of the University of Geneva.

## Abbreviation

IRB: Institutional Review Board
